# Cost-minimisation analysis of plasma exchange versus IVIg in the treatment of autoimmune neurological conditions

**DOI:** 10.1186/s12913-022-08210-z

**Published:** 2022-07-12

**Authors:** Tara Klemencic Kozul, Anna Yudina, Carley Donovan, Ashwin Pinto, Chinar Osman

**Affiliations:** 1 University Hospital Southampton NHS Foundation Trust, Southampton, UK; 2 Terumo Blood and Cell Technologies Europe, Zaventem, Belgium

**Keywords:** Plasma exchange (PLEX), Intravenous immunoglobulin (IVIg), Cost-minimisation, Autoimmune neurological disorders, Cost effectiveness

## Abstract

**Background:**

Plasma exchange (PLEX) is an effective treatment for antibody-mediated neurological disorders and has been shown to be equally efficacious to intravenous immunoglobulin (IVIg) with comparable adverse event profiles. IVIg has traditionally been the preferred treatment option due to its ease of use. However, advancing technology has allowed PLEX to be performed with a centrifugal system via peripheral access as opposed to central access via a membrane filter.

**Methods:**

We prospectively collected data from a cohort of patients who underwent PLEX at the Wessex Neurological Centre, UK, to perform a cost-minimisation analysis comparing PLEX to IVIg, the standard of care, between May 2019 and May 2020. Data obtained included indication, admission type (inpatient, daycase or intensive care), access (peripheral or central), number of PLEX cycles, exchange volume, patient weight, complications and clinical outcomes. The cost of PLEX delivered in an outpatient setting for an average 80kg person was calculated and compared to the equivalent cost of delivering IVIg by means of a cost-minimization model.

**Results:**

The provision of PLEX was roughly half as costly when compared to what it would have been for IVIg (£886 per exchange vs £1778 per infusion or £4432 per cycle of 5 exchanges vs £8890 per cycle of 5 infusions). Our cohort included a total of 44 patients who received a total of 357 PLEX exchanges during the 12-month period (the majority of which were in a daycase setting). We calculated an annual cost saving for PLEX over IVIg of £318,589. The robustness of this result was confirmed by a one-way deterministic sensitivity analysis, showing the cost-effectiveness of PLEX.

**Conclusion:**

Our findings demonstrate that PLEX is more cost-effective than IVIg in this setting. Our study supports the economic case for development of plasma exchange centres in regional neurology units, a case made all the more relevant in the context of constrained supplies of IVIg.

**Supplementary Information:**

The online version contains supplementary material available at 10.1186/s12913-022-08210-z.

## Introduction

Plasma exchange (PLEX) is a therapeutic immunological treatment whereby blood components are removed from the body and separated allowing the plasma alone to be extracted and replaced with another fluid, often human albumin solution. PLEX is an effective treatment for antibody-mediated neurological disorders. In the 1980s, it was used widely in regional neurological centres and intensive care units as first-line treatment for acute Guillain-Barre syndrome (GBS) and myasthenic crisis. However, accessibility barriers prevented its more widespread use [1, 2].

Intravenous immunoglobulin (IVIg) is a fractionated blood product consisting of concentrated immunoglobulin derived from pools of thousands of donors. It is also a treatment used for antibody-mediated neurological disorders. It is delivered as a drip, the dose-volume of which depends on the condition and weight of the patient.

There is emerging evidence of the equal efficacy of IVIg and PLEX in autoimmune neurological disorders. Two randomized trials compared the efficacy of IVIg vs PLEX in GBS; one showed that IVIg was at least as effective as PLEX in treating acute GBS [2]. The other showed that PLEX and IVIg had equal efficacy when used in the first two weeks of the disease [3]. This equal efficacy has been further confirmed in a more recent literature review [4]. There are no trials directly comparing IVIg to PLEX for the treatment of chronic inflammatory demyelinating polyneuropathy (CIDP), but there are multiple studies comparing either IVIg or PLEX to placebo, summarized in a Cochrane review, which concluded their equal efficacy for a duration of at least two to 6 weeks [5]. In the management of moderate to severe myasthenia gravis, including myasthenic crisis, two randomized trials came to the conclusion that IVIg and PLEX were equally effective [1, 6]. Moreover, treatment with PLEX led to improved clinical outcome in myasthenic patients not responsive to IVIg, as demonstrated in a retrospective review [7]. Auto-immune encephalitis, particularly anti-NMDA receptor encephalitis and voltage-gated potassium channel antibody encephalitis, have been found to respond with equal efficacy with IVIg or PLEX [8–10]. The use of PLEX as second-line therapy (where IVIg, as first line, was found to be ineffective) is recommended for conditions such as stiff person syndrome (SPS)[11–13] and acute disseminated encephalomyelitis (ADEM) [14]. The use of PLEX in neuro-myelitis optica (NMO) has been recommended by an evidence-based guideline [15].

Because PLEX and IVIg treat similar conditions, after review of the efficacy evidence, clinicians need to choose which one to use. This is influenced by ease of delivery and the side effect profile of each.

The rate of systemic reactions to IVIg is reported to be in the range of 3%–15% [16]. These reactions are often self-limiting, of mild to moderate severity, and can often be avoided by slowing down the infusion rate. IVIg also carries some rare but significant risks such as anaphylactoid reactions with reported cases of hepotitis C transmission. Since the standardization of viral inactivation steps and more in depth screening of donors, there have been no transmissions, but there is a theoretical possibility of unknown as well as novel viruses and other infectious agents becoming acquired [17].

PLEX is relatively safe, with most significant side effects originating from the method of vascular access. Central venous access carries a significantly higher risk of complications than peripheral access [18]. This risk is reduced by performing the centrifugal method via peripheral access. Removal of immunoglobulin leads to a theoretical immune compromise and an assumed increase in the risks carried by those with impaired immune systems. Citrate, the anticoagulant that is in the extracorporeal circulation during the centrifugal seperation method occasionally can cause toxicity in the form of sensory symptoms and occasionally cardiac arrhythmia. Reactions to the replacement fluids are very rare with human albumin solution.

Complications may include hemodynamic instability, sepsis and hypersensitivity to albumin for PLEX, and renal failure, hypercoagulable states and hypersensitivity to immunoglobulin for IVIg.

Studies that have compared the side effect profiles of IVIg and PLEX have previously only considered the filtration method [3]. Two studies looking at the complications experienced from PLEX found that PLEX performed by peripheral venous access carries less risk of severe complications [19, 20].

PLEX can be performed by two different techniques; centrifugation or filtration. PLEX performed in an intensive care environment is often via membrane filtration which always requires central venous access. It should be noted that the plasma extraction efficiency of membrane filtration is only 30%. In addition, the patient becomes inadvertently anticoagulated as a consequence of the heparin required to anticoagulate the extra corporeal circuit. The centrifugal method has a higher plasma extraction rate of 70% and can be delivered peripherally, and so avoids the need for high-risk vascular access. This also allows for PLEX to be delivered in a daycase setting. Furthermore, centrifugal PLEX employs citrate to anticoagulate the circuit, thus avoiding the unwanted anticoagulant effect on the patient.

IVIg is currently the standard of care for autoimmune neurological conditions because of ease of administration, thus enabling access across a range of healthcare settings. In addition, clinicians have familiarised themselves with this standard of treatment. The use of IVIg by the National Health Service (NHS) has been steadily increasing over the years despite rationing attempts. In 2018/19 NHS England spent £228 million on immunoglobulin (Ig). The volumes purchased are increasing by approximately 10% each year [21]. This has led commissioning groups in the UK to recommend a colour coding system to help local committees in prioritizing patient groups. Alternative treatments are also listed if supply runs out. The recent pandemic has affected availability of IVIg by reducing supply and increasing demand even further. IVIg is being used as a treatment for immune-mediated haematological disorders related to COVID-19 vaccinations [22]. Reserves are being kept in hospitals for these purposes alone. In addition, fewer donations have been received as people have been encouraged to stay at home.

NHS England recorded 2,514,222 g of IVIg used for neurological indications in 2018/2019 [21]. In certain conditions such as multifocal motor neuropathy (MMN), no alternative to IVIg exists [17]. However, for the majority of autoimmune neurology, PLEX is an established and safe alternative to immunoglobulins (supplementary Table 1).

We wish to re-examine the currently held doctrine that IVIg is the preferable choice of treatment for many autoimmune neurological conditions "‘on the grounds of equal therapeutic benefit, greater convenience and similar overall cost" [3]. Our cost minimisation analysis uses a real-life cohort of patients from an NHS neurology service to help illustrate the difference. In a publicly funded healthcare system such as the NHS, this issue has far-reaching implications as to how limited resources are redistributed. PLEX procedures and IVIg infusions were compared following NICE recommendations of economic evaluation of health technologies [23, 24]. Consolidated Health Economics Evaluation Reporting Standards (CHEERS) statement served as a guidance for reporting [25].

## Methods

The model for cost-minimization analysis comparing a PLEX exchange with an IVIg infusion was built in Microsoft Excel (for Office 365 MSO, 2002). Inputs for PLEX were collected during the prospective 12-month study at Wessex Neurological Centre (WNC), UK. Inputs for IVIg were obtained from the lead Clinical Advice & Immunoglobulin Pharmacist at University Hospital Southampton NHS Foundation Trust. IVIg dosing was obtained from national commissioning guidance which governs the local practice at WNC [17]. This data is available in Table 1 (for PLEX) and Table 2 (for IVIg) with appropriate references. The model is available as a supplementary material. It is set up to enable other departments worldwide to enter and calculate their costs.

**Table 1:** Model Inputs, PLEX per exchange (without Overhead Costs)

Parameter	Price per unit / hour, £	Units/ exchange	Total per exchange, £	References
**Staffing/hour**				
Consultant	122.68	0.2	24.54	NHSEmployers.org
Nurse	30.38	3	91.13	NHSEmployers.org
Admin support	13.04	0.5	6.52	NHSEmployers.org
**Capital equipment cost**			28.85	Terumo BCT price list for UK. Depends on the number of exchanges. See supplementary table 2 for calculations.
**Consumables**				
Exchange set	204.38	1	204.38	Terumo BCT price list for UK
Peripheral access, standard giving set	1.7	1	1.7	Materials Management and Distribution Unit, University Southampton Hospital
Peripheral access, blood warmer tube	2.64	1	2.64	Materials Management and Distribution Unit, University Southampton Hospital
Central access			10.27	Weighted cost calculated on the basis of the study data. See supplementary Tables 3, 4 and 5 for calculations.
**Replacement fluid and solutions**				
Albumin 5% 500 mL	42.74	8	341.92	Pharmacy Unit, University Southampton Hospital
0.9% sodium chloride 500 mL	0.8	1	0.8	Pharmacy Unit, University Southampton Hospital
Acid citrate, bag	4.6	2	9.2	Pharmacy Unit, University Southampton Hospital
Calcium, vial	12	2	24	Pharmacy Unit, University Southampton Hospital
TOTAL			**745.94**	

Data from a cohort of neurology patients receiving PLEX between May 2019 and May 2020 was prospectively collected. The data collected included indication; whether treatment was delivered electively, non-electively or on intensive care; access (peripheral or central); number of PLEX courses; exchange volume; patient weight; complications and clinical outcomes.

If PLEX had not been available at WNC between May 2019 and May 2020, the patients would have received IVIg instead. From the clinical perspective, eligibility criteria for both IVIg and PLEX are similar.

IVIg dosing was determined using NHS commissioning guidelines and confirmed by the local WNC pharmacist. The standard daily infusion of IVIg was calculated as 0.4 g/ kg. This unit of treatment was directly compared to a single PLEX exchange.

Below are outlined the breakdown of costs included in PLEX delivery:

National labour costs were obtained from NHSEmployers.org [26]. In WNC an average PLEX procedure took 3h. A band 7 (senior) nurse is required to be present throughout the procedure. 0.2h of a neurology consultant’s (supervising physician) time and 0.5h of admin support were needed

PLEX was performed on a Spectra Optia Apheresis System(Terumo Blood and Cell Technologies), a centrifugal device with the addition of a blood warmer kit. The amortization period is equal to 10 years. The service contract is free in UK for the first year.

The UK price for an exchange set was obtained from the supplier.

With regard to vascular access, where possible, the PLEX was delivered peripherally. The costs associated with peripheral access consisted of a standard giving set plus the blood warmer tube.

For some patients, the cost of central access was incurred in addition to the cost of peripheral access. In total 10 patients out of 44 had a short-term central vascular line inserted for access (vascath). A vascath is typically removed after the patient completes a treatment course of five exchanges. The cost of a centrally delivered exchange of PLEX via vascath per patient was calculated by dividing the sum cost of labour, capital equipment costs, an exchange set and the vascath by five. Two patients had a long-term central access line (apheresis line) because they were undergoing regular exchanges.

Apheresis lines are inserted by an interventional radiologist. The cost, per exchange, of PLEX delivered via apheresis line was obtained by dividing the sum cost of inserting the line, including labour, capital equipment costs, an exchange set and the apheresis line by the total number of exchanges performed using that line (29 in the case of patient 1 and 18 in the case of patient 2). This result reduced the cost of the line insertion per exchange for this type of central access (which can be used for multiple courses).

In the supplementary Table 3 we provide a full cost summary for 2 types of central access used in this study (vascath and apheresis line) that include equipment and labour costs.

Taking all of the above into account, a weighted extra cost associated with central access per patient per procedure was represented by the percentage of patients who needed vascath times the extra cost associated with vascath plus the percentage of patients who needed an apheresis line times the extra cost associated with apheresis line.

In our model it is possible to change the number of patients with each access type and the cost of various access types so it can be generalized to various scenarios/practices.

The replacement fluid was 5% human albumin. With average exchange volume being 4 L, a total of eight 500 mL bottles were needed per exchange. Two bags of anticoagulant (acid citrate) were needed for one exchange. One bag of 0.9% sodium chloride was used for each exchange. Calcium gluconate was infused to maintain plasma ionized calcium ([Ca2+]) during the procedure and thus prevent citrate reactions. Two vials per exchange were necessary. It is worth noting that in some hospitals calcium is given only in case of citrate-related complications and not as a prophylaxis.

Below are outlined the breakdown of costs included in IVIg delivery.

Direct labour costs were obtained from NHSEmployers.org. In WNC an average PLEX procedure required 5h. A band 7 (senior) nurse is required to be present throughout the procedure. 0.2h of neurology consultant time and 0.5h of admin support were required

The NHS recommendations and local WNC practice have been used to determine IVIg dose. It is equal to 0.4 g/kg per infusion. Similarly to PLEX, a course of IVIg treatment is a set of five infusions (totals 2 g per course). An average weight of 44 treated patients was 79.9 kg. Rounded up and for consistency, all IVIg calculations were based on an 80kg person.

The price that the NHS pays for Ig is renegotiated annually. This is done nationally by the Commercial Medicines Unit (CMU) and the manufacturers. It is outlined in the National Framework Agreement for Normal Human Immunoglobulins. This framework means that all NHS Trusts pay the same price for each brand of Ig and enjoy the same discount.

Because IVIg is usually delivered peripherally, the only cost incurred is that of a standard giving set. At the WNC, IVIg infusions are always done peripherally even if the patient has a central line in place.

Overhead costs have been added to the totals for PLEX and IVIG. For WNC overhead costs are represented by care group and department overheads (5.5%); trust overheads relating to direct clinical support (e.g. pharmacy, therapies) (4.9%) and trust overheads relating to general services (8.4%).

One-way deterministic sensitivity analyses have been performed to test the robustness of the results. The analyses were conducted by varying one parameter at a time (within a plausible range) while holding others constant. Our cohort extreme values (48 kg and 130 kg) and min/max confidence interval values (74 kg and 86 kg) were used to define the range for patients' weight, a major factor influencing IVIg dosing and thus cost. Given the focus of this work on an adult patient population, and the complexity of how albumin dosing is calculated according to weight for PLEX procedures, we have decided to adopt the worst case scenario of a non-variable, weight indiscriminate PLEX cost in this paper (in practice patients with smaller weight will have less plasma volume to exchange and therefore less albumin as the replacement fluid). As IVIg is commissioned centrally, publicly available information on IVIg price fluctuation over the years was used to define the range for IVIg price per gram (from £32/g on 1 July 2017 to £52 /g on 1 July of 2021). For PLEX, the number of procedures per year was varied from 50 to 1000. The range for PLEX vascular access covered 100% central access (either vascath or apheresis line) to 100% peripheral access.

## Results

**Fig. 1:**
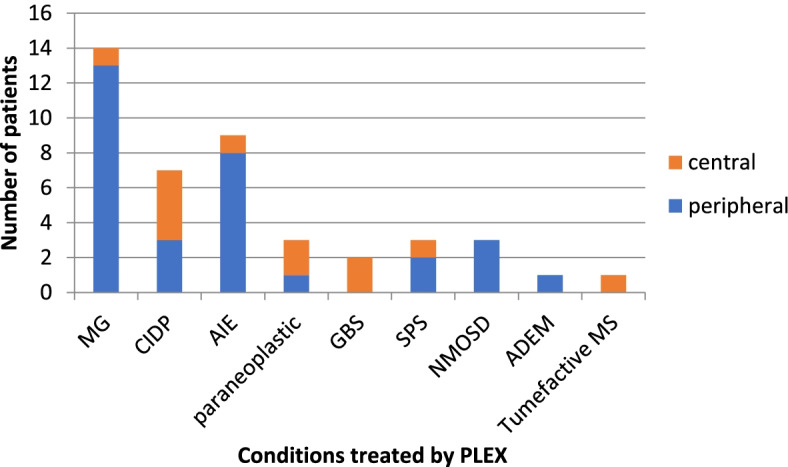
Peripheral vs central delivery of PLEX

**Fig. 2:**
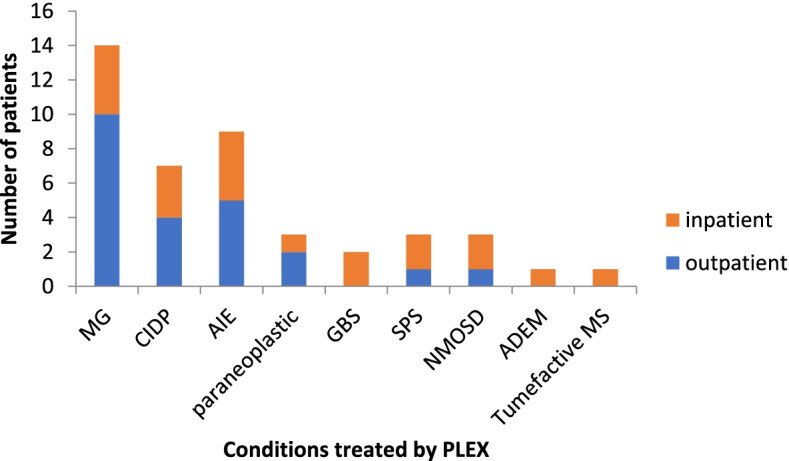
Inpatient vs daycase PLEX

We collected real-time data on patients using our PLEX service at the WNC, between May 2019 and May 2020. Forty-four patients received the treatment (patient demographics can be found in supplementary Table 7). Eight of these received repeated courses of treatments and 36 received PLEX as a one-time treatment. The number of exchanges which occurred over this period was 357. All patients received PLEX using the centrifugal Spectra Optia device. PLEX was delivered peripherally in 32 patients and centrally in 12 (Fig. 1). Nine patients had difficult peripheral access and most of those were converted to central access. Treatment was abandoned in two patients. Out of those requiring central access, two patients had apheresis lines, the other 10 had vascaths. The total number of patients receiving PLEX treatment not requiring admission was 24 (Fig. 2). The remaining 20 inpatients were either already inpatients or had to stay due to logistical reasons getting to and from their homes.

With respect to complications, three out of the patients receiving repeated courses of PLEX had minor reactions related to hypocalcaemia including paraesthesia around the mouth or extremities and hypotension. These were all easily reversible and managed. One other patient was noted to have orthostatic hypotension. Three patients were reported as unable to complete the exchange due to collapsing veins or repeated clotting of the line.

A positive outcome was achieved in 36 (82%) of the patients in our cohort. Two of the patients with unsuccessful outcomes were patients with poor peripheral access whose treatment was subsequently abandoned. Eleven (27%) of patients receiving PLEX had previously been treated with IVIg. Only one of these patients reported no therapeutic effect from either PLEX or IVIg. The conditions of the patients reporting an unsuccessful outcome included paraneoplastic syndrome, CIDP, tumefactive multiple sclerosis (MS), stiff person syndrome, NMO and MS. Several of these conditions lack clear evidence for reliably positive responses to PLEX treatment (supplementary Table 1).

The labour cost per PLEX exchange is £122.18 (£145.15 with overheads).

Machine cost per exchange depends on the number of exchanges. As 357 exchanges in total were performed in WNS daycase service, the machine cost per exchange is £28.85 (£34.28 with overheads). Detailed information can be found in Table 1.

The price for the exchange set in UK is £204.38. This can vary slightly as a function of local arrangement and the quantity purchased. The costs associated with peripheral access were £1.70 for the standard giving set plus £2.64 for the blood warmer tube thus adding £4.34 to the costs of an exchange set for patients treated peripherally. Total exchange consumable costs for peripheral access: £208.72 without Overheads (£247.96 with overheads).

Detailed information on the central access costs can be found in supplementary Tables 3, 4 and 5. We have calculated the costs of short-term central access (vascath) and long-term central access (apheresis line) separately. The vascath consumables (including vascath line, central line pack, flexi-feel ultrasound probe cover, lidocaine 1%) add up to £67.68. Vascath insertion is a 45-minute procedure that requires a neuro intensive care unit (NICU) bed, NICU consultant and an NICU nurse (of band 5 seniority or above) for additional support. The cost of labour totals £142.13. A vascath is typically inserted to last for a course of five exchanges. Therefore, for patients requiring short-term central access, the cost of a vascath line per exchange was £13.54. The additional cost to insert this vascath was £ 28.43 per exchange. The total cost for PLEX via vascath (consumables and work) was £ 41.96 per exchange (without overheads). Ten out of 44 (22.73%) patients required this access. Therefore, PLEX performed via vascath represents an additional cost of £9.54 in addition to the standard (peripheral access) exchange in WNC (without overheads).

The apheresis line consumables (including apheresis line, central line pack, lidocaine 1%) cost £126. Apheresis lines are inserted in the radiology department. The procedure normally takes 1h. The costs total £230.16 and include salaries for a consultant radiologist, two band 5 radiology nurses, a radiographer (band 6), as well as contrast medium, admin and clerical costs and the cost of using an x-ray room (supplementary Table 4). The cost per exchange of consumables is £5.67 and cost of labour is £10.36 (detailed information in Methods). The total cost to insert an apheresis line (consumables and work) was £16.03 per exchange (without overheads).

Two out of 44 (4.55%) patients in the present study had long-term apheresis lines. An apheresis line contributed an additional cost of £0.73 per exchange in addition to the standard (peripheral access) exchange at the WNC (without overheads).

Both types of central access accounted for additional £10.27 and £12.20 (without and with overheads, respectively).

The average cost of the human albumin used per exchange equals to £341.92, acid citrate £9.20, 0.9% sodium chloride £0.80, calcium gluconate £24. In total, the replacement albumin, intravenous fluids and calcium total £345.92 (£446.56 with overheads).

Detailed information about costs and quantities related to PLEX can be found in Table 1.

The labour cost per IVIg infusion is £182.94 (£217.33 with overhead costs). The IVIg cost itself per infusion was £1312.00 (£1558.66 with overhead costs). The cost for a standard giving set was £1.70 (£2.02 with overhead costs).

Detailed information about costs and quantities related to IVIg can be found in Table 2.

**Table 2:** Model Inputs, IVIg per infusion

Parameter	Price per unit, £	Units/ exchange	Total per infusion, £	References
**Staffing/ hour**				
Consultant	122.68	0,2	24.54	NHSEmployers.org
Nurse	30.38	5	151.88	NHSEmployers.org
Admin Support	13.04	0.5	6.52	NHSEmployers.org
**IVIg, g**	41	32	1312	Immunoglobulin Database; Lead Clinical Advice & Immunoglobulin Pharmacist in University Hospital Southampton NHS Foundation Trust
**Consumables**				
Peripheral access, standard giving set	1.7	1	1.7	Materials Management and Distribution Unit, University Southampton Hospital
**TOTAL**			**1496.64**	
**TOTAL with Overheads**			**1778**	

**Fig. 3:**
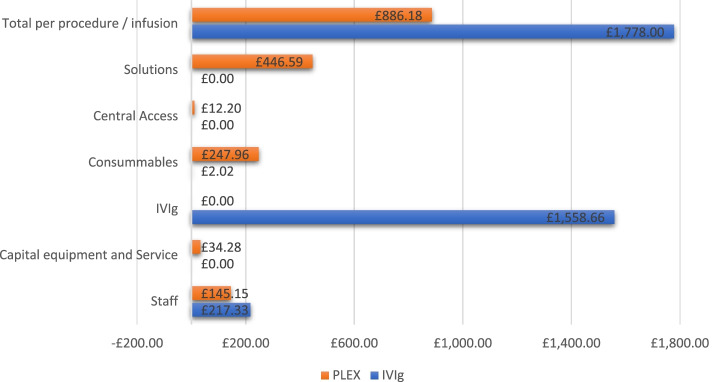
Comparative cost of PLEX and IVIg at the WNC over the 1 year period

From the perspective of a nationally funded health care system perspective (such as the NHS), the use of PLEX is roughly half as costly compared to IVIg (£886 per exchange vs £1778 per infusion or £4432 per course of five exchanges vs £8890 per course of five infusions – see Table 3). A comparative graph depicting the sub-heading costs is also available in Fig. 3. Using PLEX instead of IVIg for eligible neurological indications at the WNC between May 2019 and May 2020 resulted in over £300,000 in savings (£318,589) and would have released the use of 11,424 g of immunoglobulin to be re-routed to neurological conditions where PLEX is not effective, such as MMN or other conditions such as primary and secondary immunodeficiencies or COVID-19 related autoimmune complications.

**Table 3:** Comparative cost of PLEX and IVIg at the WNC

	PLEX	IVIg	Savings with PLEX
Cost per procedure	£886	£1778	£892
Cost per course	£4432	£8890	£4458
Cost 12 months	£316,366	£634,747	£318,589

### Sensitivity analysis

**Fig. 4:**
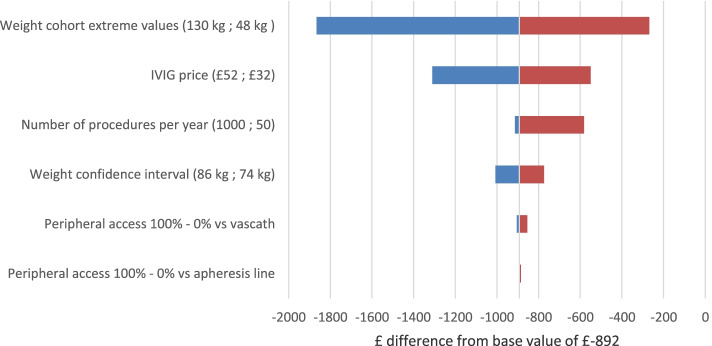
Tornado chart depicting the base case one way sensitivity analysis. The base case price difference between PLEX and IVIg procedures is -£892. The blue bars represent the parameters (y-axis) at maximum value. The red bars represent the parameters at minimum value. Actual figures used to make this chart are available in supplementary table 4

Results from a deterministic sensitivity analysis are shown in Fig. 4. The bars for all parameters stay in the negative incremental cost thus demonstrating the robustness of the base case results to plausible variation of input parameters. Patient weight is identified as a major influence on IVIg quantity and infusion costs. The break-even weight making PLEX and IVIg equal in terms of cost is 34 kg. This is assuming that the volume of albumin does not alter with weight. In practice, the volume of albumin will decrease according to the weight as well, thus providing additional cost savings for PLEX.

## Discussion

Immune-mediated neurological conditions are often treated initially with corticosteroids, IVIg or PLEX, all of which have different advantages and disadvantages. The conditions in question are often rare, so strong evidence in the form of a randomized controlled trial to either prove or disprove the efficacy of these treatments is not possible. Guidance regarding choice of treatment rests on less robust evidence such as case series and anecdotal reports. Although PLEX was shown to be effective back in the 1980s, the exchange performed exclusively via central venous access required intensive care support and was considered impractical and expensive. IVIg became more favourable due to its ease of use. The delivery of PLEX has now changed due to the development of centrifugal technology and the exchange can now be delivered peripherally.

We believe that our sample of patients is representative of a typical cohort of patients who require PLEX or IVIg treatment. We have assumed that the patient population treated with PLEX at the WNC, Southampton, UK would also be eligible for IVIg. National and international guidelines support this assumption. However in rare cases individual suitability and side effect profile drives preference for either PLEX or IVIg. This was not taken into account in this study.

In our model, we did not consider adverse effects of PLEX and IVIg. The frequency and severity of adverse events is considered similar for both PLEX and IVIg, despite the difference in event types and underlying mechanisms [27].

Adverse events are observed in 4.75 to 36% of PLEX exchanges [28–30]. Most of the effects are mild and self-limiting [20, 28]. The severe PLEX related adverse reactions can largely be attributed to central access [19, 28]. In general, at the WNC, peripheral access was used where possible. Adverse reactions reported in the World Apheresis Association registry have fallen from 11% on filtration devices (central access only) to 6% on centrifugal devices (mostly peripheral access) [30]. Moreover PLEX delivered by membrane filtration, has been associated with possible bleeding [31] and activation of complement which has not been shown to be associated with PLEX delivered via centrifugal method [32, 33].

Adverse reactions are observed in 11%–81% of patients receiving IVIg [34, 35]. The majority of these adverse reactions are easy to treat and self-limiting. Severe life-threatening side effects from IVIg are extremely rare [36, 37].

The WNC treats a catchment area of approximately 3 million. Our sample size totaled 44 patients. This relatively small patient population size is still likely to be relevant to any similarly sized regional neurological department treating immune-mediated neurological conditions.

Over the year, we believe we treated patients with a wide range of conditions, including neuroimmunological diseases with established effectiveness to PLEX as well as conditions in which the effectiveness of PLEX is less convincing (but in line with the current evidence). The majority of patients (82%) in our cohort had a successful outcome from their PLEX treatment.

As the initial capital expenditure for setting up a PLEX service is high, assessing cost-effectiveness over a short time period will favour IVIg. With time and over the course of the year, the costs involved with delivering PLEX decreased because the costs of the initial investment were spread more thinly. This also applies to the cost of the long term (apheresis) line insertions. These can be used for recurrent PLEX courses by patients returning for regular PLEX treatment. We found that using PLEX instead of IVIg for eligible neurological indications in WNC between May 2019 and May 2020 resulted in savings of over £300,000. It is worth highlighting that our cost structure is standardized nationally within the NHS framework.

Sensitivity analysis has confirmed the robustness of our results in the adult patient population treated in WNC (break-even point under the most costly assumption for PLEX is 34kg). However further investigation is necessary with respect to pediatric patients requiring less immunoglobulin. Two other major factors influencing cost-effectiveness of PLEX were IVIg price and the number of PLEX procedures per year. According to publicly available sources [38, 39] IVIg prices were either stable or growing over the past decade. PLEX remains cost-effective, even under a minimal activity of 50 procedures per year. In addition, the economies of scale result in a significant price drop per procedure with increased usage.

Other factors which may have influenced the results include vascular access, indications for treatment in our cohort and the generalisability of the model. The related cost for the adverse event profile of peripheral vs central access was not included in our model. GBS, a relatively common indication did not appear in our cohort of patients. Nevertheless, the evidence supporting treatment for GBS with either PLEX or IVIg is already well established [3]. Regarding generalisability, our model allows for corresponding adjustments, given that many variables will be different outside of the UK. The authors would be happy to hear feedback.

The global demand for IVIg has risen and yet the supplies are unable to meet this demand. Supply relies on donations of human plasma, which have most recently seen a drop during the COVID-19 pandemic. This supply and demand imbalance has led to significantly increased costs for commissioners. In a publicly funded healthcare system, this issue cannot be undermined as a driving factor to establish alternative forms of treatment. With limited resources, there is a risk that patients may not be able to continue with their IVIg treatment. It is unlikely that the supply will increase in the short-term. PLEX is an excellent alternative. Our experience at the Wessex Neurological Centre is that a day case PLEX service, delivered mainly using peripheral venous access, is able to treat patients previously stabilized on IVIg with comparable clinical outcomes and at much lower cost.

## Conclusion

Advances in PLEX technology allow treatment of patients via peripheral venous access in a daycase setting. Their outcomes and adverse event profiles are comparable to patients treated with IVIg. Analysis of data collected from neuroimmunology patients treated at our large regional neuroscience centre over the course of 1 year confirms that PLEX is more cost-effective than IVIg in this setting. Our study supports the economic case for developing a dedicated PLEX service in every regional neurology unit.

## Supplementary Information

**Additional file 1.**

**Additional file 2.**

**Additional file 3.**

**Additional file 4.**

## Data Availability

All data generated or analysed during this study are included in this published article [and its supplementary information files].
