# Supplementary material for: Cost-minimisation analysis of plasma exchange versus IVIg in the treatment of autoimmune neurological conditions
Source: BMC Health Serv Res. 2022 Jul 12;22:904. doi: 10.1186/s12913-022-08210-z (PMC9277970; doi:10.1186/s12913-022-08210-z)
Supplement: Supplementary file 2 — Additional file 2. [file 12913_2022_8210_MOESM2_ESM.docx]

| **Condition** | **PLEX** | **IVIg** |
| --- | --- | --- |
| GBS | Strong evidence  Established effective | Strong evidence  Established effective |
| CIDP | Strong evidence  Established effective | Strong evidence  Established effective for significant impairment inhibiting normal daily activities |
| Myasthenia Gravis  *Moderate to severe*  *Prethymectomy* | Strong evidence  Established effective  Weak evidence  Established effective | Strong evidence  Probably effective when sufficiently severe to require hospitalization  Moderate evidence  Probably ineffective [6] |
| Autoimmune Encephalitis  LGI1/CASPR2  NMDAR | Weak evidence  Probably effective  Weak evidence  Probably effective | Weak evidence  Possibly effective when combined with other therapies  Weak evidence  Possibly effective |
| ADEM | Weak evidence  Probably effective in corticosteroid refractory cases | Weak evidence  Possibly effective (if high-dose steroids have failed) |
| NMOSD  Acute  Maintenance | Strong evidence  Established effective (in corticosteroid refractory cases)  Weak evidence  Probably ineffective | Weak evidence  Possibly effective  Weak evidence  Probably ineffective |
| **MS**  Relapses  Progressive type | strong evidence.  Probably effective as adjuvant  Moderate evidence  Probably ineffective | Moderate evidence  Probably ineffective  Strong evidence  Probably ineffective |
| Stiff Person syndrome | Weak evidence  Possibly effective in corticosteroid refractory cases | Strong evidence  Established effective where other therapies have failed |
| Paraproteinemic polyneuropathies  IgA/IgG  IgM | Strong Evidence  Probably effective  Weak evidence  Probably ineffective | Strong evidence  Established effective for CIDP-like neuropathy  Strong evidence  Established effective only in patients with significant disability |
| Paraneoplastic neurological syndromes | weak evidence  Possibly effective | Weak evidence  Possibly effective |
| Refsum’s disease | weak evidence  Probably effective | Cannot find evidence |
| PANDAS | Moderate evidence  Probably effective | Weak evidence  Possibly effective |
| Sydenham’s chorea | Moderate evidence  Possibly effective | Weak evidence  Probably effective[7] |
| Multifocal motor neuropathy | Weak evidence  Probably ineffective | Strong evidence  Established effective |

Supplementary table 1. Comparison of the effectiveness of PLEX vs IVIg in certain neurological conditions, and the supporting evidence [1, 2]. List of conitions and accompanying PLEX column extracted from ‘Plasma Exchange in neurological disease’ [3]. Interpretation of effectiveness and evidence performed using classifications listed in EFNS and Department of Health [4, 5]

References:

1. Elovaara I, Apostolski S, van Doorn P, Gilhus NE, Hietaharju A, Honkaniemi J, van Schaik IN, Scolding N, Soelberg Sørensen P, Udd B: **EFNS guidelines for the use of intravenous immunoglobulin in treatment of neurological diseases: EFNS task force on the use of intravenous immunoglobulin in treatment of neurological diseases**. *Eur J Neurol* 2008, **15**(9):893-908.
2. Department of Health. Clinical guidelines for immunoglobulin use: update to. 2nd ed. London: Department of Health; 2011.
3. Osman C, Jennings R, El-Ghariani K, Pinto A. Plasma exchange in neurological disease. Pract Neurol. 2020;20(2):92–9.
4. Brainin M, Barnes M, Baron JC, Gilhus NE, Hughes R, Selmaj K, et al. Guidance for the preparation of neurological management guidelines by EFNS scientific task forces--revised recommendations 2004. Eur J Neurol. 2004;11(9):577–81.
5. Department of Health. Clinical Guideline for Immunoglobulin Use. 2nd ed. London: Department of Health; 2008.
6. Gamez J, Salvadó M, Carmona F, de Nadal M, Romero L, Ruiz D, et al. Intravenous immunoglobulin to prevent myasthenic crisis after thymectomy and other procedures can be omitted in patients with well-controlled myasthenia gravis. Ther Adv Neurol Disord. 2019;12:1756286419864497.
7. Boersma NA, Schippers H, Kuijpers T, Heidema J: Successful treatment of Sydenham's chorea with intravenous immunoglobulin. BMJ Case Rep 2016, 2016.

Supplementary table 2. **Labour, Capital Equipment and Service Costs for PLEX**

** For vascath, the cost per exchange is obtained by dividing by 5. For Apheresis line: from the 2 patients with this form of access one has undergone 29 exchanges and another one 18 exchanges. Thus, the cost of apheresis line consumables per exchange can be averaged as ((126/29) + (126/18))/2= £5,67. The cost of apheresis line labour per exchange can be averaged as ((230,16/29) + (230,16/18))/2= £10,36.

Supplementary table 3. Central Access Cost for PLEX

| **Apheresis line** |  |  |  |
| --- | --- | --- | --- |
| **1h procedure** |  |  |  |
| Estimates taken from RAD code 2019 workings | |  |  |
|  |  |  |  |
| Consultant radiologist |  | 1h | £ 79.80 |
| Band 5 radiology nursing x 2 |  | 1h | £ 43.20 |
| Radiographer (band 6) |  | 1h | £ 28.20 |
| Consumables | (estimated figure, remove or update if necessary) |  | £ 126.00 |
| Contrast | (estimated figure) | 1h | £ 4.00 |
| Admin and clerical |  | 1h | £ 15.60 |
| Cost of X-ray room inc. overheads |  | 1h | £ 59.36 |
| **TOTAL** |  |  | **£ 356.16** |

Supplementary Table 4. Costing summary for apheresis line insertion

| **Vascath insertion** |  |  |  |
| --- | --- | --- | --- |
| **45 min procedure** |  |  |  |
|  |  |  |  |
| GICU bed | (cost includes general nursing cost associated with GICU bed) | 45 mins | £ 74.63 |
| NEU Consultant |  | 45 mins | £ 51.30 |
| ICU Nurse (band 5), additional support | | 45 mins | £ 16.20 |
| Consumables |  |  | £ 67.68 |
| **TOTAL** |  |  | **£ 209.81** |

Supplementary Table 5. Costing summary for vascath line insertion

|  | Parameter at min value | Parameter at max value |
| --- | --- | --- |
| Peripheral access 0% - 100% vs apheresis line | -884.97 | -893.03 |
| Peripheral access 0% - 100% vs vascath | -854.17 | -904.02 |
| Weight confidence interval (74 kg; 86 kg) | -773.16 | -1006.95 |
| Number of procedures per year (50; 1000) | -581.35 | -913.86 |
| IVIG price (£32; £52) | -549.86 | -1310.18 |
| Weight cohort extreme values (48 kg - 130 kg;) | -268.54 | -1866.16 |

Supplementary table 6. Sensitivity figures used for Tornado chart

| **Gender** | **Age** | **Weight (KG)** |
| --- | --- | --- |
| F | 51 | 72 |
| F | 22 | 59 |
| M | 83 | 79 |
| M | 47 | 123 |
| M | 38 | 114 |
| F | 56 | 65 |
| M | 39 | 80 |
| F | 46 | 100 |
| F | 64 | 52 |
| M | 76 | 82 |
| M | 40 | 81 |
| F | 44 | 57 |
| M | 49 | 110 |
| F | 74 | 86 |
| M | 68 | 108 |
| F | 55 | 48 |
| M | 86 | 73 |
| M | 58 | 73 |
| M | 55 | 78 |
| F | 50 | 87 |
| F | 37 | 63 |
| M | 41 | 69 |
| M | 56 | 76 |
| F | 61 | 77 |
| M | 59 | 120 |
| M | 80 | 81 |
| M | 71 | 70 |
| F | 51 | 105 |
| F | 32 | 83 |
| F | 49 | 130 |
| F | 55 | 64 |
| M | 66 | 80 |
| F | 26 | 54 |
| M | 57 | 74 |
| M | 78 | 88 |
| M | 79 | 96 |
| M | 76 | 72 |
| M | 64 | 75 |
| F | 77 | 76 |
| F | 20 | 93 |
| F | 47 | 55 |
| M | 51 | 58 |
| F | 43 | 58 |
| M | 72 | 68 |
|  |  |  |
| **Average** | **55.7** | **79.9** |
| **SD** | **2.5** | **19.8** |
| **Upper CI (95%)** | **60.7** | **85.9** |
| **Lower CI (95%)** | **50.6** | **73.9** |

Supplementary table 7. Cohort demographics, including statistical analysis
